# C-951-01. An Analysis of the Utility of Indirect Calorimetry for Weaning *Beta* Blockade in Burn Patients

**DOI:** 10.1093/jbcr/irag033.109

**Published:** 2026-04-06

**Authors:** Briana Sylvester, Alexis Kimball, Alexandra D Davis, Anastasiya Ivanko, M Victoria P Miles, Jeffrey E Carter, Jonathan E Schoen

**Affiliations:** University Medical Center Burn Center, Louisiana State University Health Sciences Center, New Orleans, LA; University Medical Center Burn Center, Louisiana State University Health Sciences Center, New Orleans, LA; University Medical Center, Louisiana State University Health Sciences Center, New Orleans, LA; University Medical Center Burn Center, Louisiana State University Health Sciences Center, New Orleans, LA; University Medical Center, Louisiana State University Health Sciences Center, New Orleans, LA; University Medical Center Burn Center, Louisiana State University Health Sciences Center, New Orleans, LA; UCI Health Regional Burn Center, New Orleans, LA

## Abstract

**Introduction:**

Severe burn injuries induce a profound hypermetabolic state characterized by elevated resting energy expenditure (REE), increased cardiac output, and catabolic stress. Beta-blockers, such as propranolol, are commonly used to blunt this response by reducing heart rate and metabolic demands. However, criteria for safely tapering beta blockade post burn injury remain undefined. Indirect calorimetry can be used to determine a trend of the patient’s REE and the degree of their metabolic state.

**Methods:**

A prospective analysis was conducted on burn patients (11-68% Total Burn Surface Area (TBSA)) from July 2024 to July 2025 at a single, ABA-verified burn center. Patients underwent serial indirect calorimetry measurements while receiving beta-blocker therapy. REE trends were evaluated in relation to clinical parameters to assess whether normalization of metabolic rate could inform the timing of dose reduction. Secondary outcomes assessed if weight stability could be achieved by weaning propranolol based on REE to prevent significant weight loss or weight gain (+/-10%).

**Results:**

Eleven patients were included in the analysis. None of the patients in the analysis had a preexisting rate controlling medication or heart rate altering condition on their past medical history. Preliminary findings suggest that a sustained decline in REE, as measured by indirect calorimetry, appears to be related to improved clinical metabolic stability, and may serve as an objective indicator for beta-blocker weaning. There was an average weight change of +/- 3.3%, indicative of weight stability, post beta-blocker wean. Findings indicated 10-19% TBSA burn patients demonstrated an average of 2.5 days of propranolol supplementation per % TBSA prior to return to normal-for-age REE and subsequent beta-blocker discontinuation. Burns ≥20% TBSA remained on propranolol for an average 4-5 days per percent of TBSA.

**Conclusions:**

Indirect calorimetry offers an easily-implementable, valuable, and non-invasive method for monitoring metabolic recovery in burn patients. Its use may enhance clinical decision-making around beta-blocker tapering, individualizing this nuanced aspect of burn care and potentially maintaining weight stability and reducing other complications associated with inappropriate weaning.

**Applicability of Research to Practice:**

Incorporating this method into routine practice may allow for safer, more precise weaning strategies that maintain weight stability, reduce risks of metabolic imbalance, and minimize complications associated with premature or inappropriate beta-blocker discontinuation. Transitioning this research into practice could ultimately improve patient outcomes and refine evidence-based protocols in burn rehabilitation.

**Funding for the study:**

N/A.

